# Supplementary material for: A large deletion at the cortex locus eliminates butterfly wing patterning
Source: G3 (Bethesda). 2022 Jan 31;12(4):jkac021. doi: 10.1093/g3journal/jkac021 (PMC8982378; doi:10.1093/g3journal/jkac021)
Supplement: jkac021_Supplementary_Data [file jkac021_supplementary_data.docx]

**S1 Table :** Samples and accessions.

| **Accession** | **BioSample Accession** | **Sample Name** | **Phenotype** | **Inferred Genotype** | **Date Collected** | **Coverage** | **%GC** |
| --- | --- | --- | --- | --- | --- | --- | --- |
| SRR11243376 | SAMN14276602 | P1001 | Piano Keys, dark | *B/-, d/d, r/r, [ivory WT/WT]* | 30 Oct 2014 | 13.01 | 32.53 |
| SRR11243375 | SAMN14276603 | P1002 | Piano Keys, dark | *B/-, d/d, r/r, [ivory WT/WT]* | 30 Oct 2014 | 14.94 | 32.97 |
| SRR11243364 | SAMN14276604 | P1006 | Piano Keys, dark | *B/-, d/d, r/r, [ivory WT/WT]* | 23 Feb 2014 | 16.18 | 32.52 |
| SRR11243353 | SAMN14276605 | P1007 | Piano Keys, dark | *B/-, D/-, r/r, [ivory WT/WT]* | 19 Feb 2013 | 14.93 | 32.38 |
| SRR11243344 | SAMN14276606 | P1008 | Piano Keys, dark | *B/-, D/d, r/r, [ivory WT/WT]* | 19 Feb 2013 | 13.42 | 32.83 |
| SRR11243343 | SAMN14276607 | P1009 | *cydno galanthus* |  | 19 Nov 2019 | 16.14 | 32.41 |
| SRR11243342 | SAMN14276608 | P1010 | *cydno x* pale PK |  | 19 Nov 2019 | 14.07 | 32.37 |
| SRR11243341 | SAMN14276609 | P1011 | F1 pale PK x *cydno* |  | 6 Feb 2019 | 16.14 | 32.5 |
| SRR11243340 | SAMN14276610 | P1012 | F1 pale PK x *cydno* |  | 2 Feb 2019 | 16.62 | 32.6 |
| SRR11243339 | SAMN14276611 | P1013 | BC of F1 x *cydno* |  | 4 Feb 2019 | 12.49 | 32.66 |
| SRR11243374 | SAMN14276612 | P1014 | BC of F1 x *cydno* |  | 4 Feb 2019 | 14.77 | 32.19 |
| SRR11243373 | SAMN14276613 | P1015 | BC of F1 x *cydno* |  | 19 Feb 2019 | 15.93 | 32.44 |
| SRR11243372 | SAMN14276614 | P2001 | ivory | *[ivory -/-]* | 21 Jan 2019 | 15.37 | 32.24 |
| SRR11243371 | SAMN14276615 | P2002 | ivory | *[ivory -/-]* | 21 Jan 2019 | 11.69 | 32.48 |
| SRR11243370 | SAMN14276616 | P2003 | ivory | *[ivory -/-]* | 6 Jan 2019 | 8.95 | 32.45 |
| SRR11243369 | SAMN14276617 | P2004 | ivory | *[ivory -/-]* | 30 Sep 2019 | 16.11 | 32.49 |
| SRR11243368 | SAMN14276618 | P2005 | ivory | *[ivory -/-]* | 23 Sep 2019 | 16.19 | 32.74 |
| SRR11243367 | SAMN14276619 | P2006 | ivory | *[ivory -/-]* | 10 Feb 2019 | 11.47 | 33.03 |
| SRR11243366 | SAMN14276620 | P2007 | ivory | *[ivory -/-]* | 21 Jan 2019 | 15.44 | 32.77 |
| SRR11243365 | SAMN14276621 | P2008 | ivory | *[ivory -/-]* | 1 Oct 2019 | 17.40 | 32.6 |
| SRR11243363 | SAMN14276622 | P2009 | ivory | *[ivory -/-]* | 24 Jan 2019 | 12.99 | 32.64 |
| SRR11243362 | SAMN14276623 | P2010 | Piano Keys, pale | *B/-, d/d, r/r, [ivory WT/-]* | 24 Jan 2019 | 13.25 | 32.78 |
| SRR11243361 | SAMN14276624 | P2011 | ivory | *[ivory -/-]* | 20 Jun 2019 | 16.63 | 32.32 |
| SRR11243360 | SAMN14276625 | P3005 | Piano Keys, dark | *B/-, d/d, r/r, [ivory WT/WT]* | 19 Nov 2019 | 15.92 | 32.71 |
| SRR11243359 | SAMN14276626 | P3006 | Piano Keys, dark | *B/-, D/-, R/- [ivory WT/WT]* | 19 Nov 2019 | 14.44 | 32.71 |
| SRR11243358 | SAMN14276627 | P3007 | Piano Keys, dark | *B/-, D/-, R/- [ivory WT/WT]* | 16 Dec 2018 | 16.45 | 32.43 |
| SRR11243357 | SAMN14276628 | P3016 | Piano Keys, dark | *B/-, d/d, r/r, [ivory WT/WT]* | 27 Nov 2018 | 9.35 | 32.58 |
| SRR11243356 | SAMN14276629 | P3017 | Piano Keys, dark | *B/-, d/d, r/r, [ivory WT/WT]* | 14 Dec 2018 | 19.50 | 32.4 |
| SRR11243355 | SAMN14276630 | P3019 | Piano Keys, dark | *B/-, d/d, r/r, [ivory WT/WT]* | 27 Jan 2019 | 15.40 | 32.41 |
| SRR11243354 | SAMN14276631 | P4001 | Piano Keys, dark | *B/-, d/d, r/r, [ivory WT/-]* | 19 Feb 2019 | 13.24 | 32.89 |
| SRR11243352 | SAMN14276632 | P4002 | Piano Keys, pale | *B/-, D/-, R/-, [ivory WT/-]* | 19 Nov 2019 | 13.85 | 32.62 |
| SRR11243351 | SAMN14276633 | P4003 | Piano Keys, pale | *B/-, d/d, r/r, [ivory WT/-]* | 5 Feb 2019 | 13.20 | 32.48 |
| SRR11243350 | SAMN14276634 | P4004 | Piano Keys, pale | *B/-, d/d, r/r, [ivory WT/-]* | 7 Dec 2019 | 15.82 | 32.87 |
| SRR11243349 | SAMN14276635 | P4005 | Piano Keys, pale | *B/-, d/d, r/r, [ivory WT/-]* | 19 Nov 2019 | 17.01 | 32.68 |
| SRR11243348 | SAMN14276636 | P4006 | Piano Keys, pale | *B/-, D/-, R/-, [ivory WT/-]* | 19 Nov 2019 | 18.17 | 32.63 |
| SRR11243347 | SAMN14276637 | P4007 | Piano Keys, pale | *B/-, d/d, r/r, [ivory WT/-]* | 26 Jan 2019 | 9.74 | 32.77 |
| SRR11243346 | SAMN14276638 | P4008 | Piano Keys, pale | *B/-, d/d, r/r, [ivory WT/-]* | 19 Dec 2018 | 11.18 | 33.58 |
| SRR11243345 | SAMN14276639 | P4009 | Piano Keys, pale | *B/-, d/d, r/r, [ivory WT/-]* | 19 Dec 2019 | 13.96 | 32.63 |

**S1 Figure:** Dennis GWAS zoomed on Hmel218003o, showing the tip of the association peak (-log10P > 25) aligning precisely with the Dennis element designated by Morris et al (2020).


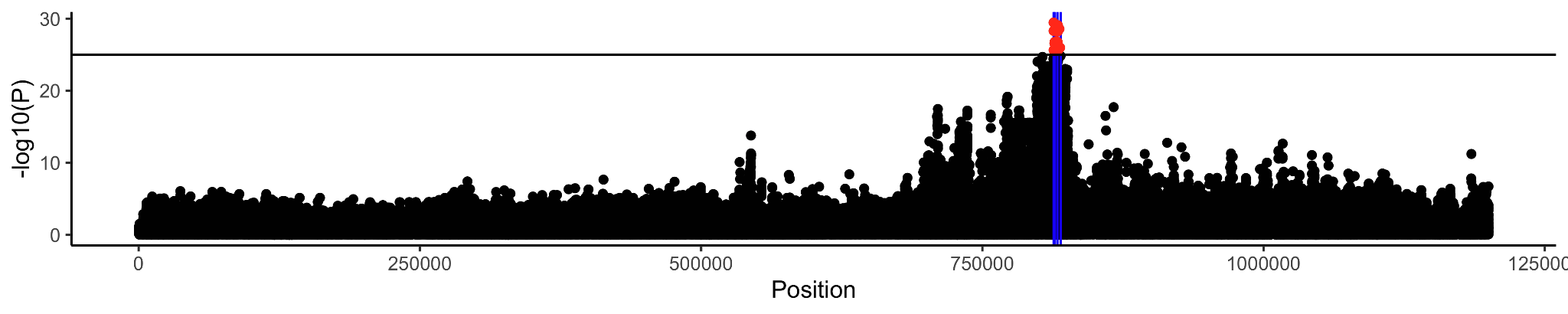


**S2 Figure:** Sashimi plot of *H. melpomene* RNA splicing at *cortex*. Ovary from Pinharanda et al 2019 (A), embryo from Dasmahapatra et al 2012 (B), pupal wings from Hanly et al 2019 (C), with 60h pupal wings above and 36h pupal wings below. Annotations of splice variants from *cortex* from the Hmel2.5 annotation are included, running in the antiparallel direction. Note that while expression of cortex is at least 20-fold higher in ovary compared to embryo or wing, no expression or splicing to the distal TSS was detected.

**
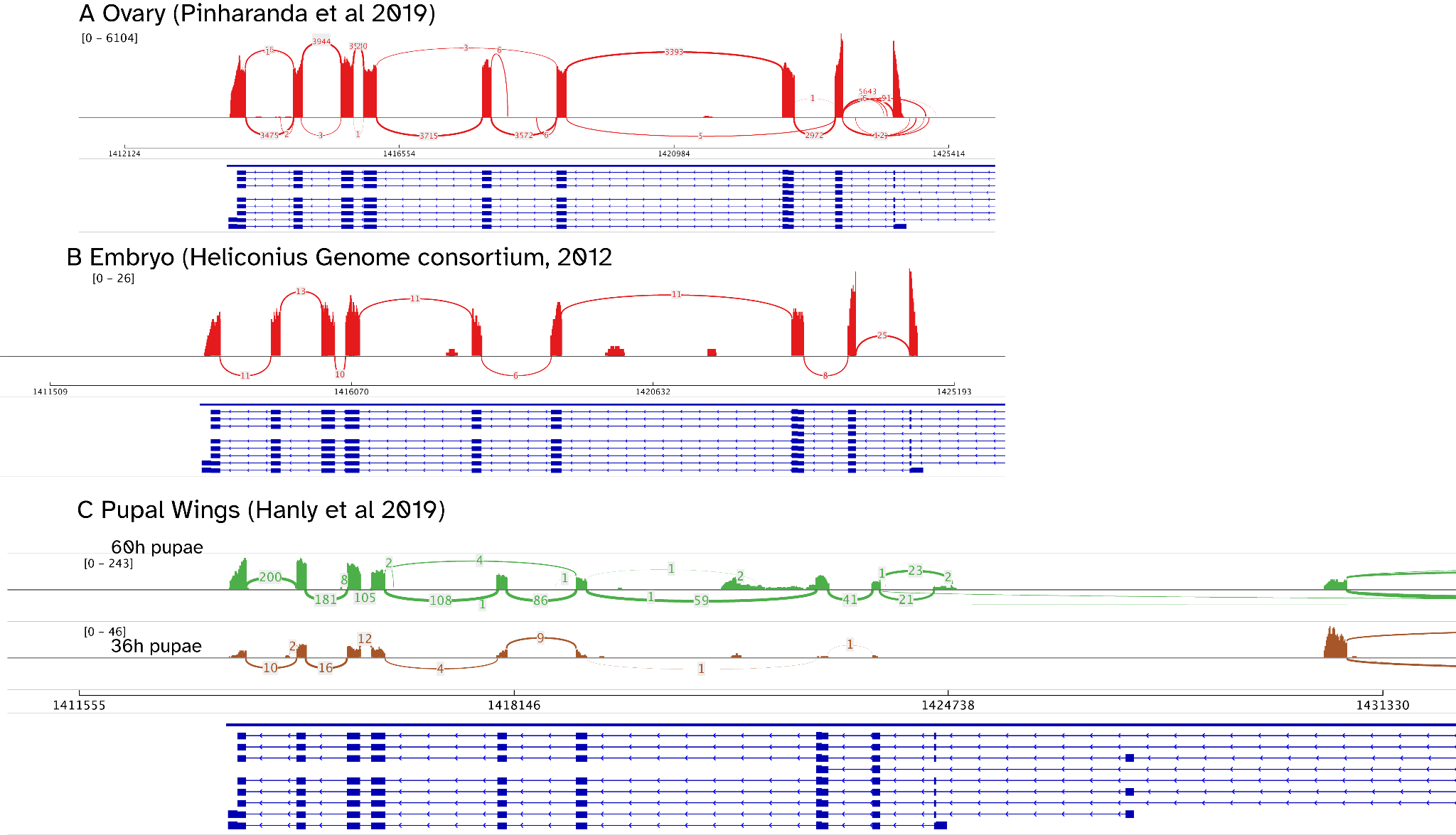
**

**S3 Figure:** Sashimi plot of *H. melpomene* RNA splicing at the full cortex annotation from larval wings, from Hanly et al 2019. The ellipsis indicates an 81kb interval. Here, the TSS at the distal promoter is used, and the proximal promoter and TSS are not transcribed.

**
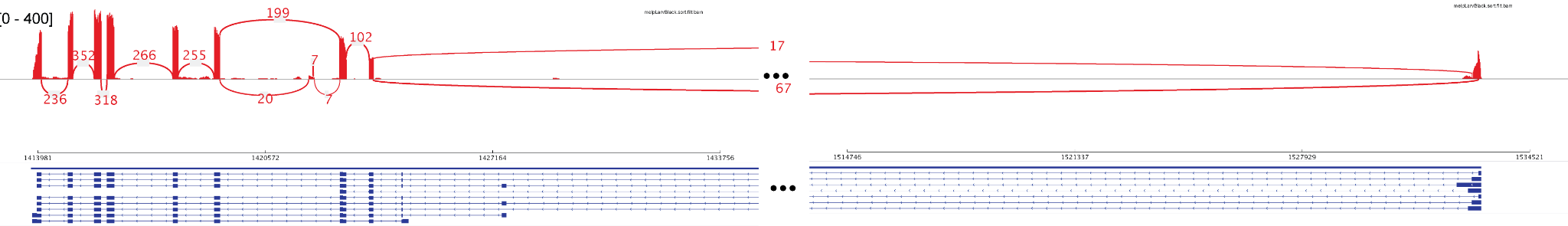
**

**S2 Table: *de novo* assembly statistics and bridging reads over the ivory deletion**

| **Sample Name** | **Phenotype** | **Assembly N50** | **scaff length, deletion 5'** | **scaff length, deletion 3'** | **scaff length, bridging over deletion** |
| --- | --- | --- | --- | --- | --- |
| P1001 | Piano Keys, dark | 14349 | 5512 bp | not detected | NA |
| P1002 | Piano Keys, dark | 16801 | 3379 bp | 5827 bp | NA |
| P1006 | Piano Keys, dark | 18351 | 5067 bp | 1616 bp | NA |
| P1007 | Piano Keys, dark | 17899 | 2988 bp | 2635 bp | NA |
| P1008 | Piano Keys, dark | 18380 | 10177 bp | 4579 bp | NA |
| P2001 | ivory | 16081 | NA | NA | 6468 bp |
| P2002 | ivory | 14164 | NA | NA | 7615 bp |
| P2004 | ivory | 21306 | NA | NA | 3716 bp |
| P2005 | ivory | 20067 | NA | NA | 5091 bp |
| P2006 | ivory | 14255 | NA | NA | 6010 bp |
| P2007 | ivory | 16831 | NA | NA | 5965 bp |
| P2009 | ivory | 26536 | NA | NA | 5250 bp |
| P2011 | ivory | 10142 | NA | NA | 9407 bp |
| P3005 | Piano Keys, dark | 17033 | 10695 bp | 5854 bp | NA |
| P3006 | Piano Keys, dark | 11943 | 6089 bp | 4475 bp | NA |
| P3007 | Piano Keys, dark | 21351 | 5183 bp | not detected | NA |
| P3016 | Piano Keys, dark | 16747 | not detected | 6013 bp | NA |
| P3019 | Piano Keys, dark | 19355 | 1549 bp | 1222 bp | NA |
| P4001 | Piano Keys, dark | 13531 | 8154 bp | not detected | NA |
| P4003 | Piano Keys, dark | 13870 | 6203 bp | not detected | NA |
| P4004 | Piano Keys, dark | 19776 | 2817 bp | not detected | NA |
| P4006 | Piano Keys, dark | 32636 | 1549 bp | 463 bp | NA |
| P4007 | Piano Keys, dark | 15523 | 7344 bp | 2679 bp | NA |
| P4008 | Piano Keys, dark | 20897 | 5920 bp | 478 bp | NA |
| P4009 | Piano Keys, dark | 13762 | 5496 bp | 3115 bp | NA |

**S3 Table: Accessions of WGS data from previous publications**

| Species and population | Group | latitude | longitude | Biosample |
| --- | --- | --- | --- | --- |
| *H. elevatus* | **silvaniform** | -5.910278 | -6.225833 | ERR053824 |
| *H. elevatus* | **silvaniform** | -6.461944 | -76.291944 | ERR053828 |
| *H. elevatus* | **silvaniform** | -5.971667 | -76.231944 | ERR053936 |
| *H. elevatus* | **silvaniform** | -6.176944 | -76.256667 | ERR1143573 |
| *H. elevatus* | **silvaniform** | -5.971667 | -76.231944 | ERR1143574 |
| *H. elevatus* | **silvaniform** | -1.075833 | -77.937222 | ERR1143575 |
| *H. melpomene meriana* | **Atlantic** | 3.9339 | 53.1258 | ERR1143603 |
| *H. melpomene meriana* | **Atlantic** | 3.9339 | 53.1258 | ERR1143604 |
| *H. pachinus* | **cydno** | 9.1206 | -79.6969 | ERR1143613 |
| *H. pachinus* | **cydno** | 9.1206 | -79.6969 | ERR1143614 |
| *H. melpomene rosina* | **West of Andes** | 9.1206 | -79.6969 | ERR260277 |
| *H. melpomene rosina* | **West of Andes** | 9.1206 | -79.6969 | ERR260278 |
| *H. melpomene rosina* | **West of Andes** | 9.1206 | -79.6969 | ERR260279 |
| *H. melpomene rosina* | **West of Andes** | 9.1206 | -79.6969 | ERR260280 |
| *H. melpomene melpomene* | **Atlantic** | 2.5222 | -51.1934 | ERR260281 |
| *H. melpomene melpomene* | **Atlantic** | 2.5222 | -51.1934 | ERR260282 |
| *H. melpomene melpomene* | **Atlantic** | 2.5222 | -51.1934 | ERR260283 |
| *H. melpomene melpomene* | **Atlantic** | 2.5222 | -51.1934 | ERR260284 |
| *H. melpomene melpomene* | **Atlantic** | 4.0833 | -52.6753 | ERR260285 |
| *H. melpomene melpomene* | **Atlantic** | 4.0834 | -52.6753 | ERR260286 |
| *H. melpomene amaryllis* | **East of Andes** | -6.096 | -76.9774 | ERR260287 |
| *H. melpomene amaryllis* | **East of Andes** | -6.096 | -76.9774 | ERR260288 |
| *H. melpomene amaryllis* | **East of Andes** | -5.6756 | -77.6747 | ERR260289 |
| *H. melpomene amaryllis* | **East of Andes** | -6.4703 | -76.3473 | ERR260290 |
| *H. melpomene aglaope* | **East of Andes** | -5.9103 | -76.2258 | ERR260291 |
| *H. melpomene aglaope* | **East of Andes** | -5.9103 | -76.2258 | ERR260292 |
| *H. melpomene aglaope* | **East of Andes** | -5.9458 | -76.2453 | ERR260293 |
| *H. melpomene aglaope* | **East of Andes** | -5.9458 | -76.2453 | ERR260294 |
| *H. cydno chioneus* | **cydno** | 9.1714 | -79.7573 | ERR260295 |
| *H. cydno chioneus* | **cydno** | 9.1714 | -79.7573 | ERR260296 |
| *H. cydno chioneus* | **cydno** | 9.1714 | -79.7573 | ERR260297 |
| *H. cydno chioneus* | **cydno** | 9.1714 | -79.7573 | ERR260298 |
| *H. timareta thelxinoe* | **timareta** |  | -82.7515 | ERR260299 |
| *H. timareta thelxinoe* | **timareta** | -6.4584 | -76.2877 | ERR260300 |
| *H. timareta thelxinoe* | **timareta** |  | -82.7515 | ERR260301 |
| *H. timareta thelxinoe* | **timareta** |  | -82.7515 | ERR260302 |
| *H. pardalinus* | **silvaniform** | -8.342222 | -74.592222 | ERR260303 |
| *H. pardalinus sergestus* | **silvaniform** | -6.477778 | -76.351667 | ERR260304 |
| *H ethilla aerotome* | **silvaniform** | -6.466667 | -76.334722 | ERR260305 |
| *H. hecale felix* | **silvaniform** | -5.971667 | -76.231667 | ERR260306 |
| *H. melpomene vulcanus* | **West of Andes** | 3.9 | -76.6325 | ERS1030540 |
| *H. melpomene vulcanus* | **West of Andes** | 3.5175 -76.757222 | | ERS1030541 |
| *H. melpomene melpomene* | **West of Andes** | 4.213 | -73.803 | ERS1030542 |
| *H. melpomene melpomene* | **West of Andes** | 5.617 | -72.3 | ERS1030543 |
| *H. melomene mocoa* | **West of Andes** | 1.178 | -76.665 | ERS1030544 |
| *H. melomene mocoa* | **West of Andes** | 1.178 | -76.665 | ERS1030545 |
| *H. melpomene malleti* | **East of Andes** | 1.813611 | -75.668611 | ERS1030546 |
| *H. melpomene malleti* | **East of Andes** | 1.609722 | -75.666944 | ERS1030547 |
| *H. melpomene malleti* | **East of Andes** | 1.750556 | -75.631944 | ERS1030548 |
| *H. melpomene melpomene* | **West of Andes** | 4.924724 | -68.925111 | ERS1030549 |
| *H. melpomene melpomene* | **West of Andes** | 4.213 | -73.803 | ERS1030550 |
| *H. melpomene melpomene* | **West of Andes** | 5.617 | -72.3 | ERS1030551 |
| *H. melpomene amandus* | **East of Andes** | -18.158333 | -63.508611 | ERS977688 |
| *H. melpomene amandus* | **East of Andes** | -18.158333 | -63.508611 | ERS977689 |
| *H. melpomene cythera* | **West of Andes** | -0.319444 | -79.336944 | ERS977691 |
| *H. melpomene cythera* | **West of Andes** | -0.319722 | -79.336944 | ERS977692 |
| *H. melomene mocoa* | **East of Andes** | 1.178056 | -76.664722 | ERS977694 |
| *H. melpomene ecuadorensis* | **East of Andes** | -4.043889 | -78.986111 | ERS977695 |
| *H. melpomene ecuadorensis* | **East of Andes** | -4.043889 | -78.986111 | ERS977696 |
| *H. melpomene plesseni x malleti* | **East of Andes** | -1.371111 | -77.874444 | ERS977697 |
| *H. melpomene malleti* | **East of Andes** | -1.036667 | -77.400833 | ERS977698 |
| *H. melpomene malleti* | **East of Andes** | -1.036667 | -77.400833 | ERS977699 |
| *H. melpomene plesseni* | **East of Andes** | -1.398056 | -78.178056 | ERS977705 |
| *H. melpomene plesseni* | **East of Andes** | -1.275833 | -78.043611 | ERS977706 |
| *H. melpomene thelxiopea* | **Atlantic** | 3.655556 | 54.039167 | ERS977708 |
| *H. melpomene vulcanus* | **West of Andes** | 8.613333 | -78.139722 | ERS977709 |
| *H. melpomene vulcanus* | **West of Andes** | 3.893333 | -76.6325 | ERS977710 |
| *H. melpomene melpomene* | **West of Andes** | 8.613333 | -78.139722 | SRR424576 |
| *H. melpomene plesseni* | **East of Andes** |  |  | SRS3676565 |
| *H. melpomene cythera* | **West of Andes** | -0.319444 | -79.336944 | SRS3676570 |
| *H. melpomene cythera* | **West of Andes** | -0.319444 | -79.336944 | SRS3676571 |
| *H. melpomene plesseni* | **East of Andes** | na | na | SRS3676572 |
| *H. melpomene rosina* | **West of Andes** | 9.1206 | -79.6969 | SRS518836 |
| *H. melpomene rosina* | **West of Andes** | 9.1206 | -79.6969 | SRS518837 |
| *H. melpomene rosina* | **West of Andes** | 9.1206 | -79.6969 | SRS518838 |
| *H. melpomene rosina* | **West of Andes** | 9.1206 | -79.6969 | SRS518839 |
| *H. melpomene rosina* | **West of Andes** | 9.1206 | -79.6969 | SRS518840 |
| *H. melpomene rosina* | **West of Andes** | 9.1206 | -79.6969 | SRS518841 |
| *H. melpomene rosina* | **West of Andes** | 9.1206 | -79.6969 | SRS518842 |
| *H. melpomene rosina* | **West of Andes** | 9.1206 | -79.6969 | SRS518843 |
| *H. melpomene rosina* | **West of Andes** | 9.1206 | -79.6969 | SRS518844 |
| *H. melpomene rosina* | **West of Andes** | 9.1206 | -79.6969 | SRS519003 |

**S4 Table: Accessions of targeted sequencing samples from previous publications**

| *Species and population* | Group | latitude | longitude | Biosample |
| --- | --- | --- | --- | --- |
| *Heliconius melpomene ssp. xenoclea* | East of Andes | -11.1745 | -75.4035 | ERR3650386 |
| *Heliconius melpomene ssp. xenoclea* | East of Andes | -11.0338 | -75.4091 | ERR3650387 |
| *Heliconius melpomene ssp.xenoclea* | East of Andes | -11.0338 | -75.4091 | ERR3650388 |
| *Heliconius melpomene ssp.xenoclea* | East of Andes | -11.0338 | -75.4091 | ERR3650414 |
| *Heliconius melpomene ssp. xenoclea* | East of Andes | -11.0338 | -75.4091 | ERR3650415 |
| *Heliconius melpomene ssp. xenoclea* | East of Andes | -11.1745 | -75.4035 | ERR3650416 |
| *Heliconius melpomene ssp. xenoclea* | East of Andes | -11.1745 | -75.4035 | ERR3650417 |
| *Heliconius melpomene ssp.xenoclea* | East of Andes | -11.0338 | -75.4091 | ERR3650418 |
| *Heliconius melpomene ssp. xenoclea* | East of Andes | -11.0364 | -75.408 | ERR3650468 |
| *Heliconius melpomene ssp. xenoclea* | East of Andes | -11.0446 | -75.4133 | ERR3650469 |
| *Heliconius melpomene ssp. Amaryllis* | East of Andes | -6.4528 | -76.2862 | ERR3650470 |
| *Heliconius melpomene ssp.amaryllis* | East of Andes | -6.4528 | -76.2862 | ERR3650471 |
| *Heliconius melpomene ssp.amaryllis* | East of Andes | -6.4528 | -76.2862 | ERR3650472 |
| *Heliconius timareta ssp. thelxinoe* | Timareta | -6.4515 | -76.2977 | ERR3650474 |
| *Heliconius timareta ssp. thelxinoe* | Timareta | -6.4515 | -76.2977 | ERR3650477 |
| *Heliconius timareta ssp. thelxinoe* | Timareta | -6.4515 | -76.2977 | ERR3650478 |
| *Heliconius timareta ssp. thelxinoe* | Timareta | -6.4519 | -76.2985 | ERR3650479 |
| *Heliconius melpomene ssp. Amaryllis* | East of Andes | -6.4567 | -76.2845 | ERR3650480 |
| *Heliconius melpomene ssp. Amaryllis* | East of Andes | -6.4555 | -76.2843 | ERR3650481 |
| *Heliconius melpomene ssp.amaryllis* | East of Andes | -6.4555 | -76.2843 | ERR3650482 |
| *Heliconius melpomene ssp. Amaryllis* | East of Andes | -6.4555 | -76.2843 | ERR3650483 |
| *Heliconius melpomene ssp. Amaryllis* | East of Andes | -6.4572 | -76.2986 | ERR3650485 |
| *Heliconius melpomene ssp.amaryllis* | East of Andes | -6.4547 | -76.2994 | ERR3650486 |
| *Heliconius melpomene ssp.amaryllis* | East of Andes | -6.4528 | -76.2862 | ERR3650487 |
| *Heliconius melpomene ssp. amaryllis* | East of Andes | -6.4572 | -76.2986 | ERR3650488 |
| *Heliconius timareta ssp. florencia* | Timareta | 1.8033 | -75.6553 | ERR3650489 |
| *Heliconius timareta ssp. thelxinoe* | Timareta | -6.4547 | -76.2994 | ERR3650490 |
| *Heliconius timareta ssp. thelxinoe* | Timareta | -6.4547 | -76.2994 | ERR3650491 |
| *Heliconius timareta ssp. florencia* | Timareta | 1.8033 | -75.6553 | ERR3650492 |
| *Heliconius timareta ssp. thelxinoe* | Timareta | -6.4547 | -76.2994 | ERR3650493 |
| *Heliconius timareta ssp. thelxinoe* | Timareta | -6.4519 | -76.2985 | ERR3650494 |
| *Heliconius timareta ssp. florencia* | Timareta | 1.7108 | -75.7089 | ERR3650495 |
| *Heliconius timareta ssp. thelxinoe* | Timareta | -6.4547 | -76.2994 | ERR3650496 |
| *Heliconius timareta ssp. thelxinoe* | Timareta | -6.4547 | -76.2994 | ERR3650497 |
| *Heliconius timareta ssp. florencia* | Timareta | 1.7108 | -75.7089 | ERR3650498 |
| *Heliconius timareta ssp. thelxinoe* | Timareta | -6.4519 | -76.2985 | ERR3650499 |
| *Heliconius timareta ssp. thelxinoe* | Timareta | -6.4519 | -76.2985 | ERR3650512 |
| *Heliconius melpomene ssp.amaryllis* | East of Andes | -6.4528 | -76.2862 | ERR3650513 |
| *Heliconius timareta ssp. florencia* | Timareta | 1.8136 | -75.6686 | ERR3650514 |
| *Heliconius melpomene ssp.amaryllis* | East of Andes | -6.4528 | -76.2862 | ERR3650515 |
| *Heliconius melpomene ssp.amaryllis* | East of Andes | -6.4528 | -76.2862 | ERR3650516 |
| *Heliconius timareta ssp. florencia* | Timareta | 1.7108 | -75.7089 | ERR3650517 |
| *Heliconius melpomene ssp. Amaryllis* | East of Andes | -6.4528 | -76.2862 | ERR3650518 |
| *Heliconius melpomene ssp.amaryllis* | East of Andes | -6.4528 | -76.2862 | ERR3650519 |
| *Heliconius melpomene ssp. plesseni* | East of Andes | -1.4371 | -78.1229 | ERR3650520 |
| *Heliconius melpomene ssp.amaryllis* | East of Andes | -6.4528 | -76.2862 | ERR3650521 |
| *Heliconius melpomene ssp. Amaryllis* | East of Andes | -6.454 | -76.3002 | ERR3650522 |
| *Heliconius melpomene ssp.plesseni* | East of Andes | -1.4371 | -78.1229 | ERR3650523 |
| *Heliconius melpomene ssp. Amaryllis* | East of Andes | -6.4537 | -76.2981 | ERR3650524 |
| *Heliconius elevatus* | elevatus | -1.1156 | -77.7783 | ERR3650525 |
| *Heliconius melpomene ssp. Amaryllis* | East of Andes | -6.4537 | -76.2981 | ERR3650526 |
| *Heliconius melpomene ssp.amaryllis* | East of Andes | -6.4537 | -76.2981 | ERR3650527 |
| *Heliconius elevatus* | silvaniform | -1.0614 | -77.6684 | ERR3650528 |
| *Heliconius melpomene ssp.amaryllis* | East of Andes | -6.4547 | -76.2994 | ERR3650529 |
| *Heliconius melpomene ssp.amaryllis* | East of Andes | -6.4537 | -76.2981 | ERR3650579 |
| *Heliconius elevatus* | silvaniform | -1.0157 | -77.5975 | ERR3650580 |
| *Heliconius melpomene ssp.amaryllis* | East of Andes | -6.453 | -76.2876 | ERR3650581 |
| *Heliconius elevatus* | silvaniform | -1.2908 | -77.8419 | ERR3650582 |
| *Heliconius melpomene ssp. Amaryllis* | East of Andes | -6.4528 | -76.2862 | ERR3650583 |
| *Heliconius melpomene ssp.amaryllis* | East of Andes | -6.4537 | -76.2981 | ERR3650584 |
| *Heliconius elevatus* | silvaniform | -1.1156 | -77.7783 | ERR3650585 |
| *Heliconius melpomene ssp.amaryllis* | East of Andes | -6.4524 | -76.2869 | ERR3650586 |
| *Heliconius elevatus* | silvaniform | -1.1878 | -77.8311 | ERR3650587 |
| *Heliconius timareta ssp. thelxinoe* | timareta | -6.4515 | -76.2977 | ERR3650588 |
| *Heliconius timareta ssp. thelxinoe* | timareta | -6.4547 | -76.2994 | ERR3650673 |
| *Heliconius timareta ssp. thelxinoe* | timareta | -6.4547 | -76.2994 | ERR3650677 |
| *Heliconius elevatus* | silvaniform | -1.1156 | -77.7783 | ERR3650679 |
| *Heliconius timareta ssp. thelxinoe* | timareta | -6.4528 | -76.2862 | ERR3650692 |
| *Heliconius elevatus* | silvaniform | -1.3382 | -77.8354 | ERR3650705 |
| *Heliconius timareta ssp. thelxinoe* | timareta | -6.4519 | -76.2985 | ERR3650708 |
| *Heliconius timareta ssp. thelxinoe* | timareta | -6.4515 | -76.2977 | ERR3650728 |
| *Heliconius timareta ssp. nov. ECU* | timareta | -1.3333 | -77.9341 | ERR3650738 |
| *Heliconius timareta ssp. thelxinoe* | timareta | -6.4528 | -76.2862 | ERR3650748 |
| *Heliconius timareta ssp. thelxinoe* | timareta | -6.4515 | -76.2977 | ERR3651361 |
| *Heliconius timareta ssp. nov. ECU* | timareta | -1.2408 | -77.9609 | ERR3651362 |
| *Heliconius timareta ssp. thelxinoe* | timareta | -6.4515 | -76.2977 | ERR3651364 |
| *Heliconius timareta ssp. nov. ECU* | timareta | -1.1156 | -77.7783 | ERR3651366 |
| *Heliconius timareta ssp. thelxinoe* | timareta | -6.4528 | -76.2862 | ERR3651367 |
| *Heliconius timareta ssp. thelxinoe* | timareta | -6.4528 | -76.2862 | ERR3651368 |
| *Heliconius timareta ssp. nov. ECU* | timareta | -1.251 | -77.6989 | ERR3651369 |
| *Heliconius timareta ssp. thelxinoe* | timareta | -6.454 | -76.3002 | ERR3651370 |
| *Heliconius timareta ssp. thelxinoe* | timareta | -6.454 | -76.3002 | ERR3651371 |
| *Heliconius melpomene ssp. Ecuadoriensis* | East of Andes | -4.0653 | -78.9587 | ERR3651374 |
| *Heliconius timareta ssp. thelxinoe* | timareta | -6.4537 | -76.2981 | ERR3651375 |
| *Heliconius timareta ssp. thelxinoe* | timareta | -6.4519 | -76.2985 | ERR3651412 |
| *Heliconius melpomene ssp.ecuadoriensis* | East of Andes | -4.0653 | -78.9587 | ERR3651415 |
| *Heliconius timareta ssp. thelxinoe* | timareta | -6.4537 | -76.2981 | ERR3651416 |
| *Heliconius timareta ssp. thelxinoe* | timareta | -6.4537 | -76.2981 | ERR3651417 |
| *Heliconius melpomene ssp. Ecuadoriensis* | East of Andes | -4.0439 | -78.9861 | ERR3651418 |
| *Heliconius melpomene ssp. malleti* | East of Andes | 1.8033 | -75.6553 | ERR3651419 |
| *Heliconius melpomene ssp. Malleti* | East of Andes | 1.8033 | -75.6553 | ERR3651426 |
| *Heliconius heurippa* | timareta | 4.175 | -73.6781 | ERR3651427 |
| *Heliconius melpomene ssp.cythera* | west | 0.1659 | -78.8882 | ERR3651428 |
| *Heliconius heurippa* | timareta | 4.175 | -73.6781 | ERR3651475 |
| *Heliconius timareta ssp. florencia* | timareta | 1.8033 | -75.6553 | ERR3651476 |
| *Heliconius timareta ssp. florencia* | timareta | 1.8033 | -75.6553 | ERR3651477 |
| *Heliconius heurippa* | timareta | 4.175 | -73.6781 | ERR3651478 |
| *Heliconius heurippa* | timareta | 4.175 | -73.6781 | ERR3651479 |
| *Heliconius heurippa* | timareta | 4.175 | -73.6781 | ERR3651482 |
| *Heliconius heurippa* | timareta | 4.175 | -73.6781 | ERR3651483 |
| *Heliconius melpomene ssp.cythera* | West of Andes | 0.1753 | -78.9075 | ERR3651484 |
| *Heliconius heurippa* | timareta | 4.175 | -73.6781 | ERR3651485 |
| *Heliconius melpomene ssp. Cythera* | West of Andes | 0.1753 | -78.9075 | ERR3651486 |
| *Heliconius heurippa* | timareta | 4.175 | -73.6781 | ERR3651487 |
| *Heliconius melpomene ssp.cythera* | West of Andes | 0.1937 | -78.8587 | ERR3651488 |
| *Heliconius heurippa* | timareta | 4.175 | -73.6781 | ERR3651489 |
| *Heliconius melpomene ssp.cythera* | West of Andes | 0.185 | -78.853 | ERR3651491 |
| *Heliconius heurippa* | timareta | 4.175 | -73.6781 | ERR3651492 |
| *Heliconius timareta ssp. florencia* | timareta | 1.7108 | -75.7089 | ERR3652193 |
| *Heliconius melpomene ssp. Cythera* | West of Andes | 0.1753 | -78.9075 | ERR3652194 |
| *Heliconius melpomene ssp.malleti* | East of Andes | 1.8033 | -75.6553 | ERR3652346 |
| *Heliconius timareta ssp. florencia* | timareta | 1.7108 | -75.7089 | ERR3652347 |
| *Heliconius melpomene ssp. Cythera* | West of Andes | 0.185 | -78.853 | ERR3652348 |
| *Heliconius melpomene ssp.malleti* | East of Andes | 1.7506 | -75.6319 | ERR3652349 |
| *Heliconius melpomene ssp.plesseni* | East of Andes | -1.4224 | -78.1729 | ERR3652350 |
| *Heliconius melpomene ssp.cythera* | West of Andes | 0.1937 | -78.8587 | ERR3652351 |
| *Heliconius melpomene ssp.malleti* | East of Andes | 1.7506 | -75.6319 | ERR3652352 |
| *Heliconius melpomene ssp.plesseni* | East of Andes | -1.4224 | -78.1729 | ERR3652353 |
| *Heliconius melpomene ssp.melpomene* | West of Andes | 4.2133 | -73.8028 | ERR3652354 |
| *Heliconius melpomene ssp. Malleti* | East of Andes | 1.7506 | -75.6319 | ERR3652355 |
| *Heliconius melpomene ssp.plesseni* | East of Andes | -1.398 | -78.1781 | ERR3652356 |
| *Heliconius melpomene ssp.melpomene* | West of Andes | 4.2133 | -73.8028 | ERR3652357 |
| *Heliconius melpomene ssp. Malleti* | East of Andes | 1.8033 | -75.6553 | ERR3652358 |
| *Heliconius melpomene ssp.plesseni* | East of Andes | -1.46 | -78.0728 | ERR3652359 |
| *Heliconius melpomene ssp.melpomene* | West of Andes | 4.0052 | -73.7775 | ERR3652360 |
| *Heliconius melpomene ssp. Malleti* | East of Andes | 1.8033 | -75.6553 | ERR3652361 |
| *Heliconius melpomene ssp. plesseni* | East of Andes | -1.46 | -78.0728 | ERR3652362 |
| *Heliconius melpomene ssp. Melpomene* | West of Andes | 4.1789 | -73.6494 | ERR3652363 |
| *Heliconius melpomene ssp. Malleti* | East of Andes | 1.8033 | -75.6553 | ERR3652364 |
| *Heliconius melpomene ssp.plesseni* | East of Andes | -1.46 | -78.0728 | ERR3652365 |
| *Heliconius melpomene ssp.melpomene* | West of Andes | 4.2133 | -73.8028 | ERR3652366 |
| *Heliconius melpomene ssp. Malleti* | East of Andes | 1.8033 | -75.6553 | ERR3652368 |
| *Heliconius melpomene ssp. ecuadoriensis* | East of Andes | -4.0653 | -78.9587 | ERR3652372 |
| *Heliconius melpomene ssp. Malleti* | East of Andes | 1.8033 | -75.6553 | ERR3652374 |
| *Heliconius melpomene ssp.melpomene* | East of Andes | 7.5878 | -72.1275 | ERR3652375 |
| *Heliconius melpomene ssp.ecuadoriensis* | East of Andes | -4.0439 | -78.9861 | ERR3652376 |
| *Heliconius cydno ssp. weymeri f. weymeri* | timareta | 3.4834 | -76.6158 | ERR3652377 |
| *Heliconius melpomene ssp. melpomene* | West of Andes | 4.2133 | -73.8028 | ERR3652378 |
| *Heliconius melpomene ssp.ecuadoriensis* | East of Andes | -4.0439 | -78.9861 | ERR3652379 |
| *Heliconius cydno ssp. weymeri f. weymeri* | timareta | 3.4834 | -76.6158 | ERR3652380 |
| *Heliconius melpomene ssp.melpomene* | West of Andes | 4.2133 | -73.8028 | ERR3652381 |
| *Heliconius melpomene ssp. Ecuadoriensis* | East of Andes | -4.0439 | -78.9861 | ERR3652382 |
| *Heliconius cydno ssp. weymeri f.weymeri* | timareta | 3.4834 | -76.6158 | ERR3652383 |
| *Heliconius melpomene ssp. melpomene* | West of Andes | 4.2133 | -73.8028 | ERR3652384 |
| *Heliconius melpomene ssp.ecuadoriensis* | East of Andes | -4.0439 | -78.9861 | ERR3652385 |
| *Heliconius cydno ssp. weymeri f.weymeri* | timareta | 3.4834 | -76.6158 | ERR3652386 |
| *Heliconius melpomene ssp. Ecuadoriensis* | East of Andes | -4.0439 | -78.9861 | ERR3652387 |
| *Heliconius melpomene ssp. Melpomene* | West of Andes | 4.0052 | -73.7775 | ERR3652388 |
| *Heliconius timareta ssp. nov. ECU* | timareta | -1.1156 | -77.7783 | ERR3652389 |
| *Heliconius timareta ssp. linaresi* | timareta | 2.6844 | -74.8881 | ERR3652390 |
| *Heliconius timareta ssp. nov. ECU* | timareta | -1.8176 | -77.9601 | ERR3652391 |
| *Heliconius timareta ssp. linaresi* | timareta | 2.6844 | -74.8881 | ERR3652392 |
| *Heliconius timareta ssp. nov. ECU* | timareta | -1.4021 | -77.7974 | ERR3652394 |
| *Heliconius timareta ssp. linaresi* | timareta | 2.6844 | -74.8881 | ERR3652395 |
| *Heliconius timareta ssp. nov. ECU* | timareta | -1.3712 | -77.8574 | ERR3652396 |
| *Heliconius melpomene ssp.melpomene* | West of Andes | 4.2133 | -73.8028 | ERR3652397 |
| *Heliconius timareta ssp. linaresi* | timareta | 2.6844 | -74.8881 | ERR3652398 |
| *Heliconius melpomene ssp.malleti* | East of Andes | 1.7108 | -75.7089 | ERR3652399 |
| *Heliconius melpomene ssp.melpomene* | West of Andes | 4.2133 | -73.8028 | ERR3652400 |
| *Heliconius timareta ssp. linaresi* | timareta | 2.6844 | -74.8881 | ERR3652401 |
| *Heliconius melpomene ssp.malleti* | East of Andes | -1.4161 | -77.729 | ERR3652402 |
| *Heliconius heurippa* | timareta | 4.175 | -73.6781 | ERR3652403 |
| *Heliconius timareta ssp. linaresi* | timareta | 2.6844 | -74.8881 | ERR3652404 |
| *Heliconius melpomene ssp. Malleti* | East of Andes | -1.1878 | -77.8311 | ERR3652405 |
| *Heliconius heurippa* | timareta | 4.175 | -73.6781 | ERR3652406 |
| *Heliconius cydno ssp. cydnides* | timareta | 3.8665 | -76.3834 | ERR3652407 |
| *Heliconius melpomene ssp. Malleti* | East of Andes | -1.2519 | -77.8196 | ERR3652408 |
| *Heliconius heurippa* | timareta | 4.175 | -73.6781 | ERR3652409 |
| *Heliconius cydno ssp. cydnides* | timareta | 3.8525 | -76.4269 | ERR3652410 |
| *Heliconius melpomene ssp.malleti* | East of Andes | -1.2519 | -77.8196 | ERR3652411 |
| *Heliconius heurippa* | timareta | 4.175 | -73.6781 | ERR3652412 |
| *Heliconius cydno ssp. cydnides* | timareta | 3.8665 | -76.3834 | ERR3652413 |
| *Heliconius melpomene ssp. Malleti* | East of Andes | -1.0983 | -77.5839 | ERR3652414 |
| *Heliconius heurippa* | timareta | 4.175 | -73.6781 | ERR3652415 |
| *Heliconius cydno ssp. cydnides* | timareta | 3.8525 | -76.4269 | ERR3652416 |
| *Heliconius melpomene ssp.vicina* | East of Andes | -4.1334 | -69.9415 | ERR3652417 |
| *Heliconius heurippa* | timareta | 4.175 | -73.6781 | ERR3652418 |
| *Heliconius cydno ssp. cydnides* | timareta | 3.8665 | -76.3834 | ERR3652419 |
| *Heliconius melpomene ssp.vicina* | East of Andes | -4.1003 | -70.0417 | ERR3652420 |
| *Heliconius heurippa* | timareta | 4.175 | -73.6781 | ERR3652421 |
| *Heliconius cydno ssp. cydnides* | timareta | 3.8665 | -76.3834 | ERR3652422 |
| *Heliconius timareta ssp. nov. 'vicina'* | timareta | -3.7701 | -70.3398 | ERR3652575 |
| *Heliconius cydno ssp. cydnides* | timareta | 3.8525 | -76.4269 | ERR3652576 |
| *Heliconius heurippa* | timareta | 3.5667 | -74.0761 | ERR3652577 |
| *Heliconius melpomene ssp.plesseni* | East of Andes | -1.4224 | -78.1729 | ERR3652579 |
| *Heliconius cydno ssp. cydnides* | timareta | 3.8525 | -76.4269 | ERR3652580 |
| *Heliconius heurippa* | timareta | 3.5667 | -74.0761 | ERR3652584 |
| *Heliconius elevatus* | silvaniform | -1.2519 | -77.8196 | ERR3652585 |
| *Heliconius cydno ssp. cydnides* | timareta | 3.8665 | -76.3834 | ERR3652586 |
| *Heliconius timareta ssp. linaresi* | timareta | 2.6844 | -74.8881 | ERR3652587 |
| *Heliconius elevatus* | silvaniform | -1.1156 | -77.7783 | ERR3652588 |
| *Heliconius cydno ssp. cydnides* | timareta | 3.8525 | -76.4269 | ERR3652589 |
| *Heliconius timareta ssp. linaresi* | timareta | 2.6844 | -74.8881 | ERR3652590 |
| *Heliconius cydno ssp. weymeri f. gustavi* | timareta | 3.4834 | -76.6158 | ERR3652591 |
| *Heliconius melpomene ssp. Malleti* | East of Andes | -1.0916 | -77.72 | ERR3652592 |
| *Heliconius timareta ssp. linaresi* | timareta | 2.6844 | -74.8881 | ERR3652593 |
| *Heliconius melpomene ssp.malleti* | East of Andes | -1.1684 | -77.7811 | ERR3652594 |
| *Heliconius cydno ssp. weymeri f.gustavi* | timareta | 2.5133 | -76.5942 | ERR3652595 |
| *Heliconius timareta ssp. linaresi* | timareta | 2.609 | -74.7776 | ERR3652596 |
| *Heliconius cydno ssp. weymeri f.gustavi* | timareta | 3.4834 | -76.6158 | ERR3652597 |
| *Heliconius melpomene ssp.malleti* | East of Andes | -1.1684 | -77.7811 | ERR3652598 |
| *Heliconius timareta ssp. linaresi* | timareta | 2.609 | -74.7776 | ERR3652599 |
| *Heliconius cydno ssp. weymeri f. gustavi* | timareta | 3.4834 | -76.6158 | ERR3652600 |
| *Heliconius melpomene ssp.malleti* | East of Andes | -1.0916 | -77.72 | ERR3652601 |
| *Heliconius timareta ssp. linaresi* | timareta | 2.6844 | -74.8881 | ERR3652602 |
| *Heliconius cydno ssp. weymeri f.gustavi* | timareta | 3.3242 | -76.6364 | ERR3652603 |
| *Heliconius timareta ssp. linaresi* | timareta | 2.6844 | -74.8881 | ERR3652604 |
| *Heliconius cydno ssp. weymeri f.gustavi* | timareta | 2.5133 | -76.5942 | ERR3652605 |
| *Heliconius timareta ssp. linaresi* | timareta | 2.6844 | -74.8881 | ERR3652606 |
| *Heliconius cydno ssp. weymeri f. gustavi* | timareta | 2.5133 | -76.5942 | ERR3652607 |
| *Heliconius timareta ssp. linaresi* | timareta | 2.6844 | -74.8881 | ERR3652608 |
| *Heliconius cydno ssp. weymeri f. gustavi* | timareta | 3.6106 | -76.5986 | ERR3652609 |
| *Heliconius cydno ssp. weymeri f.weymeri* | timareta | 3.4834 | -76.6158 | ERR3652611 |
| *Heliconius cydno ssp. weymeri f. gustavi* | timareta | 3.6106 | -76.5986 | ERR3652613 |
| *Heliconius cydno ssp. weymeri f. gustavi* | timareta | 3.6106 | -76.5986 | ERR3652615 |
| *Heliconius elevatus* | silvaniform | -1.1156 | -77.7783 | ERR3652616 |
| *Heliconius cydno ssp. weymeri f.weymeri* | timareta | 3.3242 | -76.6364 | ERR3652617 |
| *Heliconius elevatus* | silvaniform | -1.1156 | -77.7783 | ERR3652618 |
| *Heliconius cydno ssp. weymeri f.weymeri* | timareta | 3.4834 | -76.6158 | ERR3652619 |
| *Heliconius elevatus* | silvaniform | -1.1156 | -77.7783 | ERR3652620 |
| *Heliconius cydno ssp. weymeri f.weymeri* | timareta | 3.4834 | -76.6158 | ERR3652621 |
| *Heliconius elevatus* | silvaniform | -1.1878 | -77.8311 | ERR3652622 |
| *Heliconius cydno ssp. weymeri f. weymeri* | timareta | 3.4834 | -76.6158 | ERR3652624 |
| *Heliconius timareta ssp. nov. 'vicina'* | timareta | -3.8833 | -70.1881 | ERR3652626 |
| *Heliconius timareta ssp. nov. 'vicina'* | timareta | -4.0406 | -70.0997 | ERR3652628 |
| *Heliconius besckei* | silvaniform | -19.8226 | -43.6762 | ERR3652629 |
| *Heliconius besckei* | silvaniform | -20.1515 | -44.2011 | ERR3652630 |
| *Heliconius timareta ssp. nov. 'vicina'* | timareta | -3.7701 | -70.3398 | ERR3652631 |
| *Heliconius besckei* | silvaniform | -25.0462 | -51.5395 | ERR3652632 |
| *Heliconius besckei* | silvaniform | -29.4423 | -50.5798 | ERR3652633 |
| *Heliconius timareta ssp. nov. 'vicina'* | timareta | -3.7701 | -70.3398 | ERR3652634 |
| *Heliconius besckei* | silvaniform | -26.25 | -49.3836 | ERR3652635 |
| *Heliconius besckei* | silvaniform | -26.25 | -49.3836 | ERR3652637 |
| *Heliconius besckei* | silvaniform | -26.25 | -49.3836 | ERR3652638 |
| *Heliconius timareta ssp. nov. 'vicina'* | timareta | -4.1334 | -69.9415 | ERR3652639 |
| *Heliconius melpomene ssp.burchelli* | Atlantic | -6.9639 | -46.6799 | ERR3652640 |
| *Heliconius melpomene ssp.meriana* | Atlantic | 5.0818 | -54.9791 | ERR3652641 |
| *Heliconius melpomene ssp.meriana* | Atlantic | 52.1905 | -1.7037 | ERR3652642 |
| *Heliconius melpomene ssp. Burchelli* | Atlantic | -6.9639 | -46.6799 | ERR3652643 |
| *Heliconius melpomene ssp.nanna* | Atlantic | -3.8692 | -41.0192 | ERR3652644 |
| *Heliconius melpomene ssp.burchelli* | Atlantic | -6.9639 | -46.6799 | ERR3652645 |
| *Heliconius melpomene ssp.nanna* | Atlantic | -3.8692 | -41.0192 | ERR3652646 |
| *Heliconius melpomene ssp. melpomene* | West of Andes | 8.6136 | -78.1398 | ERR3652647 |
| *Heliconius melpomene ssp. nanna* | Atlantic | -15.4201 | -39.4964 | ERR3652648 |
| *Heliconius melpomene ssp.melpomene* | West of Andes | 7.6362 | -78.1897 | ERR3652649 |
| *Heliconius besckei* | silvaniform | -19.8853 | -43.6644 | ERR3652650 |
| *Heliconius besckei* | silvaniform | -19.8853 | -43.6644 | ERR3652651 |
| *Heliconius melpomene ssp.melpomene* | West of Andes | 8.6136 | -78.1398 | ERR3652652 |
| *Heliconius besckei* | silvaniform | -25.0462 | -51.5395 | ERR3652653 |
| *Heliconius melpomene ssp.melpomene* | West of Andes | 7.7568 | -77.6841 | ERR3652654 |
| *Heliconius besckei* | silvaniform | -26.25 | -49.3836 | ERR3652655 |
| *Heliconius melpomene ssp. melpomene* | West of Andes | 7.6362 | -78.1897 | ERR3652656 |
| *Heliconius pachinus* | cydno | 8.8388 | -82.7148 | ERR3652657 |
| *Heliconius pachinus* | cydno | 8.8388 | -82.7148 | ERR3652658 |
| *Heliconius pachinus* | cydno | 8.8388 | -82.7148 | ERR3652659 |
| *Heliconius pachinus* | cydno | 8.8388 | -82.7148 | ERR3652660 |
| *Heliconius pachinus* | cydno | 8.8388 | -82.7148 | ERR3652661 |
| *Heliconius pachinus* | cydno | 8.8388 | -82.7148 | ERR3653097 |
| *Heliconius pachinus* | cydno | 8.8388 | -82.7148 | ERR3653098 |
| *Heliconius pachinus* | cydno | 8.8388 | -82.7148 | ERR3653117 |
| *Heliconius melpomene ssp. meriana* | Atlantic | 52.1905 | -1.7037 | ERR3653118 |
| *Heliconius melpomene ssp.meriana* | Atlantic | 52.1905 | -1.7037 | ERR3653119 |
| *Heliconius melpomene ssp. meriana* | Atlantic | 52.1905 | -1.7037 | ERR3653120 |
| *Heliconius hecale* | silvaniform | 7.7568 | -77.6841 | ERR3653121 |
| *Heliconius hecale* | silvaniform | -6.2897 | -76.2289 | ERR3653122 |
| *Heliconius ismenius* | silvaniform | 9.1366 | -79.7236 | ERR3653123 |
| *Heliconius ismenius* | silvaniform | 9.1366 | -79.7236 | ERR3653124 |
| *Heliconius timareta ssp. timareta f. timareta* | timareta | -1.398 | -78.1781 | ERR3653125 |
| *Heliconius timareta ssp. timareta f. timareta* | timareta | -1.398 | -78.1781 | ERR3653292 |
| *Heliconius timareta ssp. timareta f.timareta* | timareta | -1.398 | -78.1781 | ERR3653293 |
| *Heliconius heurippa* | timareta | 4.175 | -73.6781 | ERR3653294 |
| *Heliconius timareta ssp. timareta f. timareta* | timareta | -1.398 | -78.1781 | ERR3653295 |
| *Heliconius timareta ssp. timareta f.timareta* | timareta | -1.398 | -78.1781 | ERR3653296 |
| *Heliconius timareta ssp. timareta f.timareta* | timareta | -1.398 | -78.1781 | ERR3653297 |
| *Heliconius timareta ssp. timareta f.contigua* | timareta | -1.398 | -78.1781 | ERR3653298 |
| *Heliconius timareta ssp. timareta f. timareta* | timareta | -1.453 | -78.107 | ERR3653299 |
| *Heliconius timareta ssp. timareta f.contigua* | timareta | -1.398 | -78.1781 | ERR3653300 |
| *Heliconius timareta ssp. timareta f.contigua* | timareta | -1.398 | -78.1781 | ERR3653301 |
| *Heliconius timareta ssp. timareta f.contigua* | timareta | -1.398 | -78.1781 | ERR3653302 |
| *Heliconius timareta ssp. timareta f. contigua* | timareta | -1.398 | -78.1781 | ERR3653303 |
| *Heliconius melpomene ssp. nanna* | Atlantic | -3.8692 | -41.0192 | ERR3653304 |
| *Heliconius timareta ssp. timareta f.contigua* | timareta | -1.398 | -78.1781 | ERR3653305 |
| *Heliconius timareta ssp. timareta f.contigua* | timareta | -1.398 | -78.1781 | ERR3653306 |
| *Heliconius timareta ssp. timareta f.contigua* | timareta | -1.398 | -78.1781 | ERR3653307 |
| *Heliconius timareta ssp. timareta f. contigua* | timareta | -1.398 | -78.1781 | ERR3653308 |
| *Heliconius timareta ssp. timareta f.contigua* | timareta | -1.398 | -78.1781 | ERR3653309 |
| *Heliconius timareta ssp. nov. ECU* | timareta | -1.1156 | -77.7783 | ERR3653310 |
| *Heliconius timareta ssp. nov. ECU* | timareta | -1.1156 | -77.7783 | ERR3653311 |
| *Heliconius timareta ssp. nov. ECU* | timareta | -1.1156 | -77.7783 | ERR3653392 |
| *Heliconius melpomene ssp. nanna* | Atlantic | -3.8692 | -41.0192 | ERR3653393 |
| *Heliconius timareta ssp. nov. ECU* | timareta | -1.2908 | -77.8419 | ERR3653394 |
| *Heliconius timareta ssp. nov. ECU* | timareta | -1.2908 | -77.8419 | ERR3653395 |
| *Heliconius timareta ssp. nov. ECU* | timareta | -1.1878 | -77.8311 | ERR3653396 |
| *Heliconius timareta ssp. nov. ECU* | timareta | -1.3333 | -77.9341 | ERR3653397 |
| *Heliconius timareta ssp. nov. ECU* | timareta | -1.251 | -77.6989 | ERR3653398 |
| *Heliconius timareta ssp. nov. ECU* | timareta | -1.251 | -77.6989 | ERR3653399 |
| *Heliconius melpomene ssp.nanna* | Atlantic | -3.8692 | -41.0192 | ERR3653400 |
| *Heliconius timareta ssp. nov. ECU* | timareta | -1.251 | -77.6989 | ERR3653401 |
| *Heliconius timareta ssp. nov. ECU* | timareta | -1.1156 | -77.7783 | ERR3653402 |
| *Heliconius timareta ssp. nov. ECU* | timareta | -1.1878 | -77.8311 | ERR3653403 |
| *Heliconius melpomene ssp.nanna* | Atlantic | -3.8692 | -41.0192 | ERR3653404 |
| *Heliconius timareta ssp. nov. ECU* | timareta | -1.1156 | -77.7783 | ERR3653405 |
| *Heliconius timareta ssp. nov. ECU* | timareta | -1.1156 | -77.7783 | ERR3653406 |
| *Heliconius timareta ssp. nov. ECU* | timareta | -1.2908 | -77.8419 | ERR3653407 |
| *Heliconius melpomene ssp.nanna* | Atlantic | -3.8692 | -41.0192 | ERR3653408 |
| *Heliconius melpomene ssp.malleti* | East of Andes | -1.251 | -77.6989 | ERR3653409 |
| *Heliconius melpomene ssp. malleti* | East of Andes | -1.0614 | -77.6684 | ERR3653410 |
| *Heliconius melpomene ssp. Malleti* | East of Andes | -1.0614 | -77.6684 | ERR3653411 |
| *Heliconius melpomene ssp.malleti* | East of Andes | -1.1684 | -77.7811 | ERR3653412 |
| *Heliconius melpomene ssp. Malleti* | East of Andes | -1.1684 | -77.7811 | ERR3653413 |
| *Heliconius melpomene ssp.malleti* | East of Andes | -1.1684 | -77.7811 | ERR3653414 |
| *Heliconius melpomene ssp.malleti* | East of Andes | -1.1156 | -77.7783 | ERR3653415 |
| *Heliconius melpomene ssp.malleti* | East of Andes | -1.1156 | -77.7783 | ERR3653416 |
| *Heliconius melpomene ssp. Malleti* | East of Andes | -1.1156 | -77.7783 | ERR3653417 |
| *Heliconius melpomene ssp.malleti* | East of Andes | -1.1156 | -77.7783 | ERR3653419 |
| *Heliconius melpomene ssp. Malleti* | East of Andes | -1.1156 | -77.7783 | ERR3653420 |
| *Heliconius melpomene ssp. malleti* | East of Andes | -1.1156 | -77.7783 | ERR3653421 |
| *Heliconius melpomene ssp.malleti* | East of Andes | -1.1156 | -77.7783 | ERR3653422 |
| *Heliconius melpomene ssp.malleti* | East of Andes | -1.1156 | -77.7783 | ERR3653423 |
| *Heliconius melpomene ssp.malleti* | East of Andes | -1.0614 | -77.6684 | ERR3653424 |
| *Heliconius melpomene ssp. Malleti* | East of Andes | -1.0614 | -77.6684 | ERR3653425 |
| *Heliconius melpomene ssp.malleti* | East of Andes | -1.0614 | -77.6684 | ERR3653427 |
| *Heliconius melpomene ssp.malleti* | East of Andes | -1.0614 | -77.6684 | ERR3653428 |
| *Heliconius melpomene ssp. Malleti* | East of Andes | -1.0614 | -77.6684 | ERR3653429 |
| *Heliconius melpomene ssp.malleti* | East of Andes | -1.1156 | -77.7783 | ERR3653430 |
| *Heliconius melpomene ssp.ecuadoriensis* | East of Andes | -4.0439 | -78.9861 | ERR3653529 |
| *Heliconius melpomene ssp.meriana* | Atlantic | 3.6883 | -54.0825 | ERR3653532 |
| *Heliconius melpomene ssp.meriana* | Atlantic | 3.6883 | -54.0825 | ERR3653533 |
| *Heliconius melpomene ssp.nanna* | Atlantic | -19.0984 | -40.1862 | ERR3653534 |
| *Heliconius timareta ssp.linaresi* | timareta | 2.609 | -74.7776 | ERR3653535 |
| *Heliconius pachinus* | cydno | 8.8388 | -82.7148 | ERR3653536 |
| *Heliconius timareta ssp. timareta f.timareta* | timareta | -1.398 | -78.1781 | ERR3653537 |
| *Heliconius timareta ssp. timareta f.timareta* | timareta | -1.398 | -78.1781 | ERR3653539 |
| *Heliconius timareta ssp. timareta f. contigua* | timareta | -1.398 | -78.1781 | ERR3653543 |
| *Heliconius melpomene ssp.amaryllis* | East of Andes | -6.4528 | -76.2862 | ERR3656073 |
| *Heliconius melpomene ssp. plesseni* | East of Andes | -1.4224 | -78.1729 | ERR3656232 |
| *Heliconius melpomene ssp. vicina* | East of Andes | -4.1334 | -69.9415 | ERR3656233 |
| *Heliconius melpomene ssp. Cythera* | West of Andes | 0.185 | -78.853 | ERR3656234 |
| *Heliconius cydno ssp. weymeri f.weymeri* | cydno | 3.4834 | -76.6158 | ERR3656235 |
| *Heliconius timareta ssp. linaresi* | timareta | 2.6844 | -74.8881 | ERR3656282 |
| *Heliconius timareta ssp. linaresi* | timareta | 2.6844 | -74.8881 | ERR3656283 |
| *Heliconius timareta ssp. linaresi* | timareta | 2.609 | -74.7776 | ERR3656288 |
| *Heliconius melpomene ssp. melpomene* | West of Andes | 4.2133 | -73.8028 | ERR3656289 |
| *Heliconius heurippa* | timareta | 4.175 | -73.6781 | ERR3656290 |
| *Heliconius melpomene ssp. melpomene* | West of Andes | 4.2133 | -73.8028 | ERR3656291 |
| *Heliconius melpomene ssp. Malleti* | East of Andes | 1.7108 | -75.7089 | ERR3656292 |
